# Enhancing Entomological Surveillance to Combat *Aedes*‐Borne Diseases in Southern Iran

**DOI:** 10.1155/jotm/8874426

**Published:** 2026-07-17

**Authors:** Fatemeh Bagheri, Abdoljabbar Zakeri, Hossein Farshidi, Mahmoud Hosseinpour, Kaveh Soleimani, Yousef Salari, Jafar Hatami, Masoud Yaryan, Fatemeh Nikpour, Abdolreza Miroliaei, Ahmad Raeisi, Ahmadali Enayati, Mohammad Mehdi Sedaghat, Ghobad Moradi, Madineh Abbasi, Saideh Yousefi

**Affiliations:** ^1^ Department of Diseases Control, Hormozgan Health Center, Hormozgan University of Medical Sciences, Bandar Abbas, Iran, hums.ac.ir; ^2^ Social Determinants in Health Promotion Research Center, Research Institute for Health, Hormozgan University of Medical Sciences, Bandar Abbas, Iran, hums.ac.ir; ^3^ Cardiovascular Research Center, Rajaie Cardiovascular Institute, Tehran, Iran; ^4^ Organ Donation Center, Hormozgan University of Medical Sciences, Bandar Abbas, Iran, hums.ac.ir; ^5^ Department of Diseases Control, Bandar Lengeh Health Center, Hormozgan University of Medical Sciences, Bandar Lengeh, Iran, hums.ac.ir; ^6^ Institute for Environmental Research, Tehran University of Medical Sciences, Tehran, Iran, tums.ac.ir; ^7^ Department of Vector-Borne Diseases, Centre for Communicable Diseases Control, Ministry of Health, Tehran, Iran, behdasht.gov.ir; ^8^ Department of Vector Biology and Control of Diseases, School of Public Health, Tehran University of Medical Sciences, Tehran, Iran, tums.ac.ir; ^9^ Department of Medical Entomology and Vector Control, Mazandaran University of Medical Sciences, Sari, Iran, mazums.ac.ir; ^10^ Centre for Communicable Diseases Control, Ministry of Health, Tehran, Iran, behdasht.gov.ir; ^11^ Social Determinants of Health Research Center, Tabriz University of Medical Sciences, Tabriz, Iran, tbzmed.ac.ir; ^12^ Infectious and Tropical Diseases Research Center, Tabriz University of Medical sciences, Tabriz, Iran, tbzmed.ac.ir; ^13^ Department of Public Health, Sirjan School of Medical Sciences, Sirjan, Iran; ^14^ Student Research Committee, Sirjan School of Medical Sciences, Sirjan, Iran

**Keywords:** *Aedes*, chikungunya, dengue fever, entomological surveillance, Zika

## Abstract

**Background:**

Vector‐borne diseases, particularly those transmitted by *Aedes* mosquitoes, including dengue, chikungunya, Zika, and yellow fever, represent significant public health challenges globally. The rapid spread of these diseases in urban environments, driven by climate change and human activities, necessitates effective surveillance and control strategies. In Iran, the emergence of *Aedes aegypti* has raised concerns about potential outbreaks of the disease, particularly in southern regions such as Hormozgan Province.

**Methods:**

A retrospective descriptive study was conducted using routinely collected entomological surveillance data from the national invasive *Aedes* surveillance program of Iran CDC. The study covered urban areas of Bandar Abbas County, southern Iran, from January 2022 to December 2025. Biweekly ovitrap surveillance and larval survey data were analyzed to describe temporal trends of egg, house, container, and Breteau indices. Surveillance outputs during full and reduced operational capacity phases were compared descriptively. Associations between indices and meteorological variables were assessed using Pearson or Spearman correlation analyses.

**Results:**

Entomological indices showed marked temporal and interannual variability between 2022 and 2025. Egg indices varied seasonally, with an obvious peak in April 2023. House, container, and Breteau indices generally declined during 2023, coinciding with intensified surveillance and control activities, but resurged in parts of 2024 following a substantial reduction in operational capacity. During the reduced‐capacity phase, egg indices remained nonzero, whereas larval indices were predominantly zero, likely reflecting decreased surveillance coverage. No statistically significant correlations were observed between entomological indices and temperature, relative humidity, or rainfall.

**Discussion and Conclusion:**

The findings emphasize the need for continuous entomological surveillance and community engagement in managing *Aedes*‐borne diseases. This study highlights the necessity for integrated vector management strategies that include community‐based interventions and robust monitoring systems. Despite initially low entomological indices, effective surveillance and control measures are essential for preventing *Aedes*‐borne disease outbreaks in southern Iran. The insights gained can guide public health policies and serve as a model for similar regions facing vector‐borne disease threats.

## 1. Introduction

Vector‐borne diseases, particularly those transmitted by *Aedes* mosquitoes, such as dengue fever (DF), chikungunya, Zika, and yellow fever, pose significant global health threats [1–3]. The rapid spread of these diseases presents a considerable public health challenge and contributes to substantial economic burdens in various countries [4, 5]. Despite the approval of a dengue vaccine for individuals with prior dengue immunity, which demonstrates modest efficacy, there remains an urgent need for effective therapeutics and vaccines to reduce morbidity and alleviate the overall disease burden [6]. Due to its preference for feeding on human blood and its high adaptability to urban areas, *Aedes aegypti* is the primary vector for several important arboviruses [7]. This mosquito primarily bites during the day and twilight [8]; however, its flight distance is short and it does not disperse far from its larval habitats. Nevertheless, its high adaptability to urban environments complicates conventional control measures [9]. Human activities significantly influence the spread of mosquito vectors, particularly through the movement of people and goods. Additionally, climate change, urban development, and environmental degradation further impact the distribution of invasive *Aedes* species [10].

Effective management of mosquito‐borne diseases relies on accurately identifying the mosquito species responsible for transmitting pathogens [11]. In regions lacking effective vaccines, ongoing vector surveillance and control are essential for DF prevention [12]. Various techniques have been developed for monitoring mosquito populations, focusing primarily on early life stages such as eggs, larvae, and pupae [13, 14]. Effective vector surveillance can prevent or mitigate outbreaks in their early stages [15]. While common entomological indices like the Breteau index (BI), container index (CI), and house index (HI) provide insights into infestation levels, they often fall short in accurately estimating adult populations and true transmission risks.

Since 2016, the surveillance program for *Aedes*‐borne diseases, encompassing both human and entomological health aspects, has been incorporated into the framework of the Iran Disease Management Center [16]. This program focuses on monitoring international entry points for the presence of invasive *Aedes* mosquitoes.

Entomological surveillance plays a crucial role in the systematic and continuous collection, analysis, and interpretation of data aimed at planning, implementing, and evaluating vector control operations. Specifically, the surveillance of invasive *Aedes* species is an essential component of integrated vector management (IVM), supporting evidence‐based policymaking for the prevention and control of diseases such as DF, chikungunya, and Zika. The objectives of this program include the early detection of invasive *Aedes* mosquitoes entering new geographical areas, assessing their potential for spread, evaluating the risk of disease transmission to humans, and identifying primary breeding sites. Furthermore, the program assesses the effectiveness of vector control interventions and determines insecticide susceptibility levels. Three potential scenarios based on the presence and abundance of invasive *Aedes* mosquitoes have been defined, each requiring tailored operational plans. The first scenario involves the absence of invasive *Aedes*; the second pertains to localized establishment in the early stages; and the third indicates widespread establishment. In response to the third scenario, the health system must evaluate the dynamics of the mosquito population, including distribution, larval ecology, population density, and seasonal trends, alongside the cost‐effectiveness and efficacy of vector control programs, as well as the viral contamination of mosquitoes collected during disease outbreaks.

In 2019, coinciding with the COVID‐19 crisis, reports of *Ae. aegypti* were documented in Bandar Lengeh County, located in Hormozgan Province. Consequently, the entomological surveillance approach transitioned from monitoring international entry points to comprehensive citywide surveillance, including house‐to‐house inspections.

By 2021, *Ae*. *aegypti* was also reported in Bandar Abbas County and several other counties. This study aims to evaluate the effectiveness of the entomological surveillance program, following reports of *Ae. aegypti* in Bandar Abbas County. Insights gained from this experience could serve as a valuable model for other regions in the country or worldwide. Moreover, this study intends to identify shortcomings in the current program, providing recommendations for policymakers at the ministry level. Such improvements are essential for enhancing the program’s responsiveness to the challenges posed by *Aedes*‐borne disease outbreaks.

## 2. Methodology

### 2.1. Study Area

**FIGURE 1:**
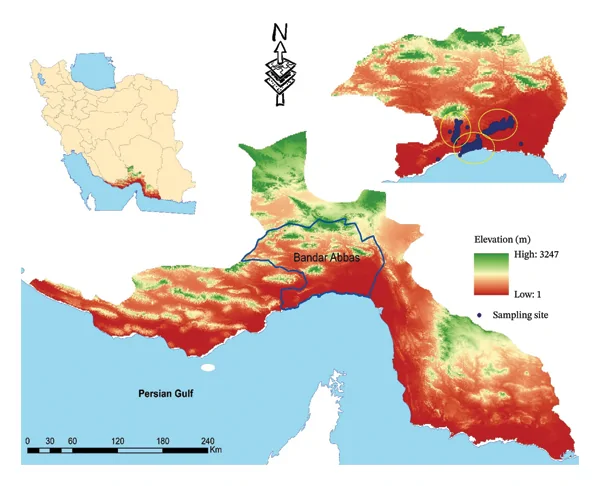
Geographical map of the study area, Bandar Abbas County, Hormozgan Province, southern Iran.

Bandar Abbas is a coastal city located in Hormozgan Province, southern Iran, positioned at approximately 27°11′ N latitude and 56°17′ E longitude. It serves as the capital city and is situated along the Persian Gulf, covering an area of around 1000 km^2^ (Figure 1). The climate of this area is classified as subtropical, characterized by hot and humid conditions, as one of the hottest cities in Iran during the summer months, with an average annual temperature of approximately 28°C. The extended hot season lasts about nine months, starting in early March and peaking in July and August, followed by a brief cool season beginning in early December, influenced by cooler air masses from the west. The economy of Bandar Abbas is heavily reliant on its bustling ports, which facilitate significant passenger and cargo traffic. The presence of these busy ports contributes to the city’s role as a vital trade hub in the region. Many residents are employed in maritime industries, trade, and logistics, while others engage in fishing and tourism‐related activities. The region’s high humidity levels, particularly during the summer, can exacerbate the heat, making it feel even warmer. This combination of factors influences the lifestyle and occupational choices of the local population, as they adapt to the challenging climatic conditions and the economic opportunities presented by the port activities.

### 2.2. Study Design

This study was conducted between January 2022 and December 2025 in Bandar Abbas County, Hormozgan Province, southern Iran. The study used a retrospective descriptive design based on routinely biweekly collected entomological surveillance data from the national invasive *Aedes* surveillance program implemented by the Iran Centers for Disease Control and Prevention (CDC).

The surveillance activities were carried out exclusively in urban areas. The study did not involve active field sampling by the authors; instead, data were gathered through oviposition trap (ovitrap) surveillance and larval surveys were analyzed. The primary objective was to describe temporal trends in entomological indices and to assess variations in surveillance outputs in relation to operational capacity and selected environmental variables.

### 2.3. Organization of the Entomological Surveillance Program

**FIGURE 2:**
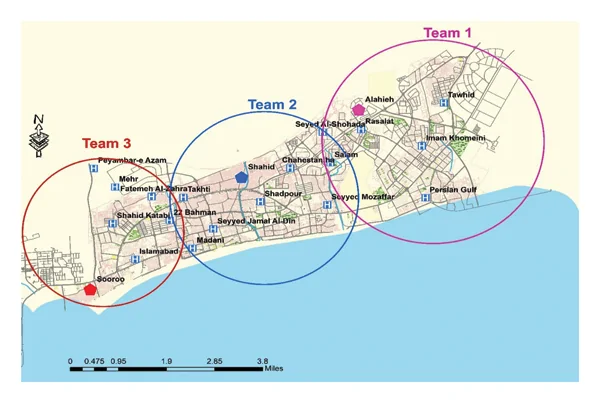
Entomological surveillance team zones in Bandar Abbas County, southern Iran, 2022–2025.

**FIGURE 3:**
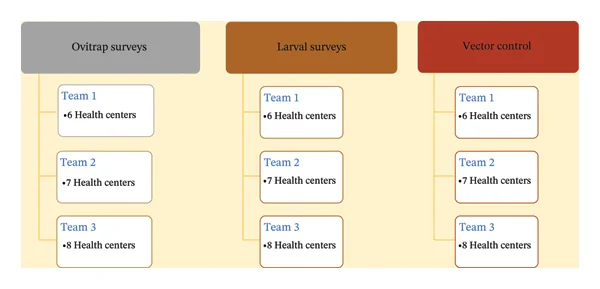
Invasive *Aedes* mosquito surveillance program in Bandar Abbas County, southern Iran, 2022–2025.

The entomological surveillance program for invasive *Aedes* mosquitoes in Bandar Abbas County was organized according to the national surveillance protocol of the Iran CDC. The county was divided into three operational zones, each coordinated by a designated health center. In each zone, surveillance activities were supervised by an entomologist responsible for planning, implementation, and quality control (Figures 2 and 3). From 2022 to mid‐2024, a total of 57 trained personnel were involved in surveillance activities across the three zones. Surveillance was conducted biweekly, with approximately 900 ovitraps deployed and 260 households inspected for larval presence during each surveillance round. Following August 2024, the operational capacity of the program was substantially reduced. This reduced‐capacity phase continued through December 2025. The number of personnel decreased to 9, resulting in a reduction of ovitraps deployed biweekly to 300 and larval inspections to 100 households.

### 2.4. Sampling Strategy and Site Selection

Sampling was conducted using a systematic random sampling approach. Urban areas within each operational zone were divided into predefined parts, and sampling locations were selected at regular spatial intervals to ensure representative geographic coverage. For ovitrap surveillance, site selection was preceded by environmental assessment and source reduction activities to ensure suitability for the presence of potential *Aedes* breeding habitats. Ovitraps were placed only in locations with environmental conditions conducive to natural oviposition, such as shaded areas near human dwellings, vegetation, or structures that could support mosquito activity. This approach aimed to maximize the sensitivity of ovitrap surveillance and reduce false‐negative findings.

## 3. Data Collection

The following methods were used for collecting the data.

### 3.1. Entomological Surveillance

#### 3.1.1. Ovitrap Surveillance

Ovitrap surveillance was conducted in accordance with the national *Aedes* surveillance protocol. Black plastic containers with a capacity of 1.5–2 L were used as ovitraps. A small hole was created near the upper edge of each container to prevent overflow during rainfall. The containers were filled with water to approximately one‐third of their volume and placed in fixed locations near human dwellings in shaded and low‐disturbance environments, such as along walls or near vegetation, at a height of less than 1 m above ground level.

To enhance oviposition attractiveness, fermented infusion was prepared by soaking 100 g of dry straw in 10 L of water for 1 week. After fermentation, the straw was removed, and the infusion was used in the ovitraps. Rough‐surfaced oviposition papers were placed along the inner walls of the ovitraps to facilitate egg laying. Each paper was labeled with the ovitrap identification number and date.

**FIGURE 4:**
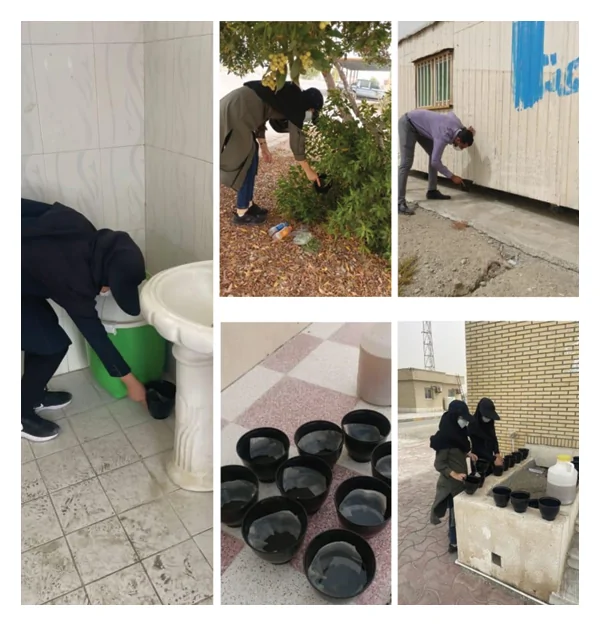
Ovitrap setting in Bandar Abbas county, southern Iran, 2022–2025.

Ovitraps were inspected every 15 days during the mosquito activity season. Oviposition papers were collected after 3–5 days, stored in moist conditions, and transported to the laboratory for analysis. Eggs were counted using a magnifying device, and the average number of eggs per ovitrap per 24 h was calculated. Species identification was performed by hatching eggs under laboratory conditions and identifying fourth‐instar larvae using a standard morphological identification key [17]. Data from the ovitraps were utilized to assess the presence and seasonal variations of vector populations (Figure 4).

#### 3.1.2. Larval Surveys

**FIGURE 5:**
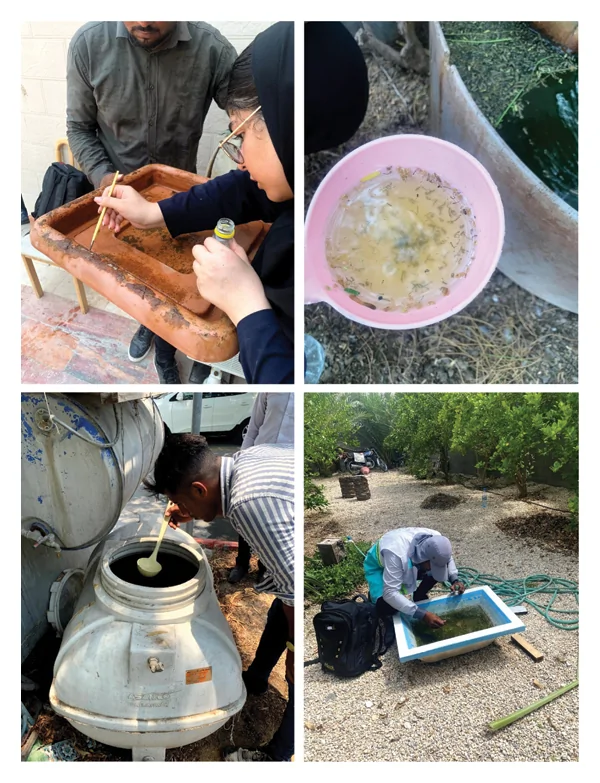
Larval sampling from different habitats in Bandar Abbas county, southern Iran, 2022–2025.

Larval surveys were conducted biweekly during the mosquito activity season. Potential larval habitats were inspected in residential areas, including water storage containers, discarded tires, and other artificial containers. Large containers were sampled using a standard dipper, while larvae from smaller containers were collected using droppers or by transferring water into white trays (Figure 5).

Collected larvae were preserved in lactophenol or transported alive for rearing to the fourth instar prior to identification. Species identification was performed using standard morphological identification keys. Data from larval surveys were used to calculate standard entomological indices and to map positive and negative breeding sites for operational planning and control activities.

### 3.2. Entomological Indices

The following standard entomological indices were calculated.

- Egg index: mean number of eggs per ovitrap per 24 h.
- HI: percentage of houses infested with larvae or pupae.
- CI: percentage of water‐holding containers infested with larvae or pupae.
- BI: number of positive containers per 100 houses inspected.

### 3.3. Data Analysis

Entomological *Aedes* surveillance data were entered into Microsoft Excel for cleaning and preliminary organization. Statistical analyses were performed using IBM SPSS Statistics (IBM Corp., Armonk, NY, USA), Version 24. Descriptive statistics were used to summarize entomological indices, including mean, standard deviation, and temporal trends of the egg index, HI, CI, and BI across the study period.

To assess the impact of operational capacity on entomological surveillance outcomes, the study period was divided into two operational phases: Phase I (January 2022–July 2024), representing full operational capacity, during which 57 personnel were involved and approximately 900 ovitraps were deployed biweekly; and Phase II (August 2024–December 2025), representing reduced operational capacity, following a reduction in human resources to 9 personnel and a decrease in ovitrap deployment to approximately 300 units per biweekly interval. Differences in entomological indices between the two phases were evaluated using the Mann–Whitney *U* test.

Normality of continuous variables was assessed using the Shapiro–Wilk test. As entomological indices were not normally distributed, nonparametric tests were applied. Differences in entomological indices across the three study years (2022–2025) were assessed using the Kruskal–Wallis H test.

Meteorological data were obtained from the Bandar Abbas Meteorological Office. Temporal associations between entomological indices and meteorological variables, including temperature, relative humidity, and rainfall, were evaluated using correlation analyses. Pearson correlation coefficients were calculated for normally distributed variables, while Spearman’s rank correlation coefficients were used when normality assumptions were not met. Statistical significance was set at *p* < 0.05 for all analyses. All statistical tests were two‐sided.

Geographic mapping and spatial visualization of the study area, operational zones, and health centers were performed using ArcGIS software (ESRI, Redlands, CA, USA), Version 10.5. The geographic coordinates of health centers and surveillance units were obtained from the routine records of the local health system. These coordinates were used to generate maps illustrating the spatial distribution of surveillance zones, ovitrap deployment areas, and larval survey coverage.

## 4. Results

### 4.1. Overview of Entomological Surveillance Outcomes (2022–2025)

**FIGURE 6:**
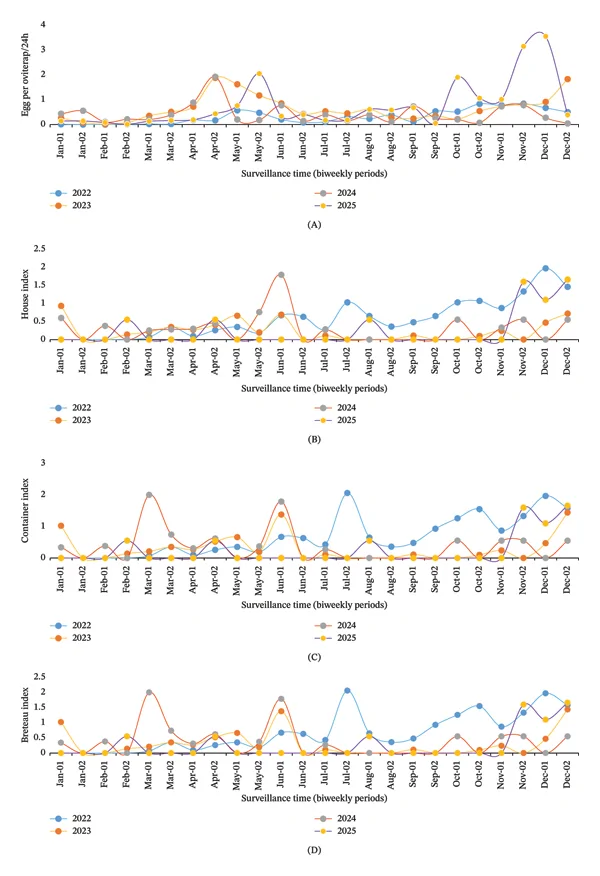
Entomological indices trend in Bandar Abbas County, southern Iran, 2022–2025. (A) Egg, (B) house, (C) container, and (D) Breteau indices.

Entomological surveillance conducted in Bandar Abbas County between January 2022 and December 2025 generated longitudinal data on four standard indices: egg index, HI, CI, and BI. Monthly variations and interannual trends of these indices are presented in Figure 6.

### 4.2. Temporal Trends of Entomological Indices

#### 4.2.1. Egg Index

Over the 4‐year period (2022–2025), the egg index demonstrated clear seasonal and interannual variability. While the peak during 2022–2024 was predominantly observed in April, with the highest value recorded in April 2023 (1.88), a shift in the temporal pattern was observed in 2025, when higher egg indices occurred mainly during the autumn and early winter months, with the maximum value recorded in December 2025 (3.55).

#### 4.2.2. HI

The HI showed a mean value of approximately 0.3 ± 0.3, ranging from 0.0 to 1.97 across the 4‐year study period (2022–2025). Yearly trends demonstrated a gradual increase during the second half of 2022, with notable peaks observed in November and December. In 2023, the HI exhibited two distinct peaks in January and December. In 2024, a resurgence in household‐level infestation was observed, particularly in January and June, coinciding with warmer conditions. In 2025, overall HI values remained low for most months, yet a clear increase was recorded in late autumn and early winter, with peak values in November (1.6) and December (1.66). Across the 4 years, the magnitude and timing of these peaks varied substantially, indicating persistent interannual variability in household‐level mosquito breeding activity (Figure 6B).

#### 4.2.3. CI

The overall mean CI across the 4‐year study period (2022–2025) was approximately 0.4, with a maximum value of 3.14 recorded in July 2022. Interannual analysis revealed that the mean CI was highest in 2022 (approximately 0.78), followed by substantially lower mean values in 2023 and 2024 (approximately 0.26 and 0.29, respectively), and the lowest mean value observed in 2025 (approximately 0.20). Peak CI values were observed in July 2022, June 2023, and March 2024, indicating marked variability in both the magnitude and timing of container positivity across years. In 2025, although the overall CI remained low for most months, sporadic increases were observed during late autumn and early winter, particularly in November and December (Figure 6C).

#### 4.2.4. BI

The average BI across the 4‐year study period (2022–2025) was approximately 0.4, with a maximum value of 2.06 recorded in July 2022. Interannual comparison showed that the mean BI was highest in 2022 (0.67), followed by lower mean values in the subsequent years, including 2023 (0.30), 2024 (0.38), and the lowest mean value observed in 2025 (0.25). Peak BI values occurred in July and December during 2022, June and December in 2023, and March and June in 2024. In 2025, although overall BI values remained low for most months, distinct increases were observed in late autumn and early winter, with peak values recorded in November and December (Figure 6D).

Kruskal–Wallis analysis revealed statistically significant differences across the four study years (2022–2025) for the CI, HI, and BI (*p* < 0.05), whereas differences in the egg index were not statistically significant (*p* = 0.059). The egg index exhibited its highest mean rank in 2023, although interannual variation did not reach statistical significance. In contrast, CI, HI, and BI showed their highest mean ranks in 2022, followed by a decline in 2023 and 2024 and a further reduction in 2025, indicating a significant decrease in larval and household infestation indicators over time (Table 1).

**TABLE 1:** Comparison of entomological indices across three years using the Kruskal–Wallis test in Bandar Abbas County, southern Iran, 2022–2025.

Index	Year	*N*	Mean rank	Kruskal–Wallis H	df	*p* value
Egg index	2022	24	39.17	7.439	3	0.059
	2023	24	60.71			
	2024	24	47.06			
	2025	24	47.06			

Container index	2022	24	67.23	18.514	3	< 0.001^∗^
	2023	24	45.25			
	2024	24	46.56			
	2025	24	34.96			

House index	2022	24	63.92	12.931	3	0.005^∗^
	2023	24	46.44			
	2024	24	46.92			
	2025	24	36.73			

Breteau index	2022	24	63.46	12.679	3	0.005^∗^
	2023	24	45.56			
	2024	24	48.21			
	2025	24	36.77			

*Note:* Higher mean rank indicates higher index values.

^∗^Statistically significant difference (*p* < 0.05).

### 4.3. Comparison of Entomological Indices Between Operational Phases

The results of the phase‐wise comparison of entomological indices are presented in Table 2. The Mann–Whitney *U* test showed no statistically significant difference in the egg index between Phase I and Phase II (*U* = 978.0, *Z* = −0.582, *p* = 0.560). In contrast, significant differences were observed for larval indices, with CI, HI, and BI being significantly lower during Phase II compared with Phase I (CI: *U* = 628.0, *Z* = −3.409, *p* = 0.001; HI: *U* = 693.0, *Z* = −2.890, *p* = 0.004; BI: *U* = 704.5, *Z* = −2.818, *p* = 0.005).

**TABLE 2:** Comparison of entomological indices between surveillance phases using the Mann–Whitney *U* test in Bandar Abbas County, southern Iran, 2022–2025.

Index	Phase	*N*	Mean rank	Mann–Whitney U	*Z*	*p* value
Egg index	Phase I	62	49.73	978	−0.582	0.56
	Phase II	34	46.26			

Container index	Phase I	62	55.37	628	−3.409	0.001^∗^
	Phase II	34	35.97			

House index	Phase I	62	54.32	693	−2.89	0.004^∗^
	Phase II	34	37.88			

Breteau index	Phase I	62	54.14	704.5	−2.818	0.005^∗^
	Phase II	34	38.22			

*Note:* Values are based on biweekly intervals. Differences between phases were assessed using the Mann–Whitney *U* test.

^∗^Statistically significant difference (*p* < 0.05).

### 4.4. Metrological Data

**FIGURE 7:**
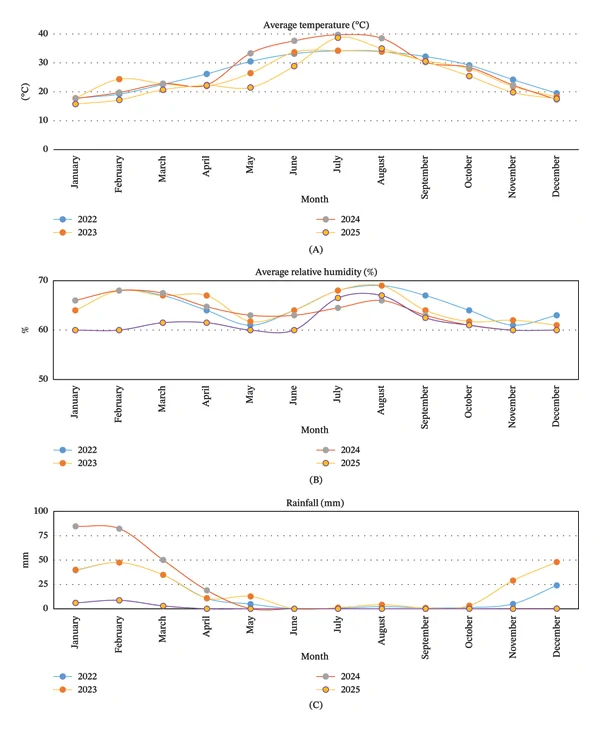
Monthly meteorological data by year, Bandar Abbas County, Southern Iran, 2022–2025. (A) Average temperature (°C), (B) average relative humidity (%), and (C) rainfall (mm).

The average monthly temperature exhibited a clear seasonal pattern, with the lowest values consistently observed during winter months and peak temperatures occurring in summer (Figure 7A). The highest average temperature was recorded in July 2024 (39.8°C), followed closely by July 2025 (38.8°C), indicating intensified summer heat during the later years of surveillance. In contrast, the lowest average temperatures were observed in January 2025 (15.8°C) and February 2025 (17.2°C), as well as December 2024 (17.3°C). Interannual variability was evident, with 2023 showing comparatively lower temperatures during several winter and spring months, while 2024 and 2025 were characterized by markedly higher summer temperatures. Overall, these findings suggest an increasing intensity of summer temperatures in the most recent years of the study period.

Average relative humidity showed a consistent seasonal pattern throughout the 4‐year study period (2022–2025), with higher values generally observed during summer months and lower values during late autumn and winter (Figure 7B). The highest relative humidity recorded across all years was 69%, occurring in August 2022 and August 2023, whereas the lowest values (60%) were observed in November and December 2024. Interannual comparisons indicated that relative humidity patterns in 2023 closely resembled those of 2022, although slight decreases were noted during several months. In contrast, 2024 exhibited a comparatively more stable range of humidity values, particularly during the summer months. Overall, relative humidity varied within a narrow range across the study period, suggesting limited interannual variability compared with temperature.

Monthly rainfall exhibited strong seasonal variation during the study period (2022–2025), with precipitation largely concentrated in winter and early spring months, while several summer and early autumn months showed little to no rainfall (Figure 7C). The highest monthly rainfall was recorded in January 2024 (84.7 mm), representing the maximum value observed throughout the study period. Interannual comparisons revealed that 2024 experienced markedly higher rainfall levels, particularly during the early months of the year (January–March), compared with the other years. In contrast, rainfall in 2022 and 2023 showed greater variability but generally remained within a lower range, while 2025 was characterized by minimal precipitation. Overall, rainfall patterns were highly seasonal, with pronounced concentration in the early months of the year.

Correlation analysis between meteorological variables and entomological indices in Bandar Abbas showed predominantly weak associations (Table 3). Pearson correlation analysis indicated no statistically significant relationships between average temperature or rainfall and the entomological indices (*p* > 0.05). A weak negative correlation was observed between relative humidity and the egg index (*r* = −0.361, *p* = 0.012); however, this association was not confirmed by Spearman’s rank correlation analysis and therefore was not considered robust.

**TABLE 3:** Correlation analysis of meteorological data and entomological indices in Bandar Abbas, southern Iran, 2022–2025.

Index	Variables	Pearson correlation	*p* value	*N*
Egg	Average temperature (°C)	−0.215	0.142	48
	Relative humidity (%)	−0.361	0.012^∗^	48
	Rainfall (mm)	−0.152	0.304	48

Container	Average temperature (°C)	−0.042	0.777	48
	Relative humidity (%)	0.06	0.684	48
	Rainfall (mm)	0.003	0.986	48

House	Average temperature (°C)	−0.103	0.488	48
	Relative humidity (%)	−0.14	0.344	48
	Rainfall (mm)	−0.059	0.688	48

Breteau	Average temperature (°C)	−0.083	0.575	48
	Relative humidity (%)	−0.027	0.856	48
	Rainfall (mm)	0.01	0.948	48

^∗^Statistically significant difference (*p* < 0.05).

## 5. Discussion

DF is one of the most important arbovirus diseases and is transmitted through the bite of *Aedes* mosquitoes [18]. *Aedes aegypti*, as the main vector of the disease, can adapt to urban environments, come into contact with humans, and transmit the disease, especially in tropical and subtropical zones of the world [19]. This species was first reported from Iran in 2020 and is currently established in Hormozgan, Sistan and Baluchistan, and Bushehr Provinces, located in the south, southeast, and southwest of the country, respectively [20]. So, Iran is known as a high‐risk area for DF outbreaks due to the presence of mosquito vectors, favorable climate conditions, and its border with disease‐infected countries [21–23]. Before 2024, no local transmission of DF had been reported in Iran. However, in 2024, the first evidence of local dengue transmission emerged in the southern parts of the country, and subsequent reports indicated a gradual expansion of locally acquired cases across several areas along the southern coastal area of Iran [22, 24, 25]. After this outbreak, DF is considered one of the major public health problems in Iran, and so the World Health Organization [26] is collaborating with Iran’s Ministry of Health and Medical Education (MoHME) to enhance host and vector surveillance efforts as the main strategies for controlling DF [18, 22]. *Aede*s surveillance programs based on entomological indicators play an important role in the risk warning, provision of quality intervention programs, and the evaluation of control operations [27, 28].

The result of the present study is in agreement with the study conducted in Pakistan, which revealed that *Ae. aegypti* was active throughout the year, with the highest frequency during summer and autumn [29]. In a study conducted in Saudi Arabia, the high abundance of this species was recorded during both winter and spring seasons and rarely during summer [30]. And also, in another study conducted in western Saudi Arabia, the peak activity of *Ae. aegypti* was reported in winter [31]. However, the weather circumstances and ecological factors among various regions are different, and this can influence the seasonal activity of mosquitoes.

Rian, temperature, and relative humidity are very important factors that can affect the mosquitoes’ breeding activities [32]. The results of our study showed that there was no significant relationship between meteorological factors and the abundance of *Ae. Aegypti,* which is consistent with the studies conducted in Indonesia that showed no correlation between climatic factors and the presence of immature stages of *Ae. aegypti* [33, 34]. But our result is different from the study conducted by Al‐Azab et al., in Saudi Arabia, who reported that meteorological factors are a very important component of the spatial and temporal distribution of DF vectors [35].

As mentioned earlier *Ae. aegypti* was reported for the first time in Iran in 2020, and after that, the MoHME carried out extensive control programs to control the mosquito population, and it seems that these control methods, with appropriate quantity and quality, have played an important role in reducing the *Ae. aegypti* population and entomological indices despite the appropriate climatic conditions.

There are many methods for collecting various stages of *Aedes* mosquitoes for surveillance programs. Methods for capturing the adult stage of *Ae. aegypti* include adult traps (ADT) that collect gravid *Ae. aegypti* searching for oviposition sites; BG‐sentinel (BGS) traps, which use an attractant such as artificial human skin odor to capture host‐seeking mosquitoes; CDC‐light traps (CDC) that use CO_2_ and UV light to attract host‐feeding mosquitoes; indoor (ASP‐I) and outdoor (ASP‐O) aspirators, which are battery‐powered; and human landing catch (HLC), which is one of the most efficient surveillance methods for anthropophilic mosquitoes such as *Ae. aegypti* [36].

There are methods for capturing pre‐adult stages, such as the ovitrap and the mosquito oviposition trap (MOT). These are easy‐to‐use tools suitable for assessing the presence of *Aedes* mosquitoes in a given area [37, 38]. Considering that each trap has its advantages and disadvantages for collecting mosquitoes at certain life stages; therefore, integrated surveillance methods should be employed as an effective tool for monitoring *Ae. aegypti* in each region [36].

Despite the importance of adult stage surveillance in accurately predicting the epidemic trend of *Aedes*‐borne diseases [39], in this study, due to limitations in human and financial resources, we could not implement adult surveillance programs.

CI, HI, BI, and egg index are among the main indices used for identifying the high‐risk areas of DF [40], predicting disease outbreaks, and formulating appropriate control measures in high‐risk regions that *Ae. aegypti* has been established [41]. During the study period, the highest rates of CI, HI, and BI were recorded in 2022. In 2023, due to the intensity of IVM with sufficient human resources, entomological indices such as CI, HI, and BI decreased; subsequently, in 2024, these indices increased, potentially due to reductions in both the quality and quantity of entomological surveillance programs owing to decreased human resources. These changes coincided with the DF outbreak reported in the region during that period and may have contributed to an increased risk of transmission [24]. The DF outbreak resulted in an increase in local inhabitants’ knowledge and attitude about the vector’s ecology, leading them to contribute to the elimination of breeding sites. Consequently, in 2025, the aforementioned indices reached their lowest levels during the study period.

However, cryptic breeding sites still exist, and the elimination of visible breeding places may shift mosquito populations to these cryptic habitats, potentially influencing control programs [42, 43]. As mentioned earlier, the CI, HI, BI, and egg index are among the primary indices used for identifying high‐risk areas for DF. Among these, the HI and BI are particularly important because they are related to positive container habitats within human dwellings [31].

Regarding DF transmission risk, the Pan American Health Organization categorizes it into three levels: low (HI < 0.1%), medium (HI = 0.1%–5%), and high (HI > 5%) [28, 43]. According to this classification, the dengue transmission risk in the study area was medium during some months, indicating that this area is at high risk for DF transmission.

However, based on WHO criteria, dengue transmission risk is categorized as follows: no risk of transmission (BI < 5), low risk of transmission (5 ≤ BI < 10), medium risk of transmission (10 ≤ BI < 20), and high risk of transmission (BI ≥ 20) [44].

Totally, in this study, HI and BI indices were below a critical level based on the criteria of WHO [45]. According to some studies, there is no significant correlation between CI, HI, and BI with the occurrence of DF [28, 40, 46, 47]. In contrast, in several studies conducted in Southeast Asia, including Indonesia, Vietnam, and India, there was a positive correlation between entomological indices and dengue outbreaks [48–50]. Entomological indices in some ancient foci of DF, such as Indonesia, Malaysia, and Pakistan, are higher than those in Iran [28, 29, 34, 51, 52].

As mentioned earlier, Iran is a new focus of DF and *Ae. aegypti* was introduced in this country in 2020. Therefore, it is expected that vector population and related indices in Iran will be lower compared to the old foci of the diseases.

In addition, the existence of a lot of cryptic aquatic habitats that cannot be visually detected, which can affect the quantity of entomological indices, should also be taken into account.

However, in 2012, following the introduction of *Ae. aegypti* into Mecca City, Saudi Arabia, the rates of the HI, CI, and BI differed between wet and dry seasons. In both circumstances, these indices were higher than those observed in our study area [31]. In another study conducted by Xiaobo Liu between 2016 and 2019 in China, involving three classes of areas, Class I with several DF outbreaks in past years, Class II areas with reported indigenous DF cases or relatively high DF risk, and Class III areas with reported imported DF cases and evidence of *Aedes* distribution, the results showed that the index values in Class I and II areas were higher than in our study area. Generally, the indices in Class I areas were higher than in our study area most of the time [37].

Some studies have shown that there is no correlation between entomological indices and dengue transmission [42, 43]. It appears that these indices are influenced by human resources and the quality and quantity of their activities. Furthermore, as previously mentioned, the ability of health workers to find cryptic breeding sites can also affect the rate of these indices.

IVM is the most effective in reducing CI, HI, and BI compared to other control methods [53]. Alternative methods like oviposition traps and egg density indices may provide better assessment of infestation rates than conventional indices [54]. Overall, effective dengue vector control requires a community‐based, integrated approach tailored to local eco‐epidemiological and sociocultural settings [53].

There is notavailable specific treatment or vaccine for DF, so IVM remains the best method for effective prevention and control of DF. Health education about the breeding places of *Ae. aegypti*, environmental management, continuous monitoring of the vector population, and researching the ecology and biology of *Ae. aegypti* can be effective in preventing widespread DF outbreaks in the southern Iran [53].

In addition to entomological surveillance, significant supplementary measures are being implemented in the study area to enhance community engagement and awareness. House‐to‐house care teams conduct direct face‐to‐face training sessions with residents, emphasizing the importance of eliminating larval breeding sites. Moreover, extensive educational campaigns are disseminated across all available social media platforms, as well as through local media outlets, to raise awareness about the removal of mosquito breeding habitats. Alongside these initiatives, neighborhood surveys are organized based on block mapping to identify nonresidential breeding sites, complementing the entomological programs. This region benefits from a well‐coordinated teamwork approach, with effective intrasectoral and intersectoral collaboration involving local municipalities, environmental agencies, military and security forces, and the judiciary. Regular meetings are held to identify high‐risk areas within the city, and collaborative efforts are made to address these issues, especially when there are gaps in financial and human resources. Such collaborations have significantly fostered community participation and established a cohesive health program in the district, contributing to the reduction of larval indices. The experience gained from entomological assessments, combined with strong intra‐ and intersectoral cooperation, public education, and awareness‐raising initiatives, can serve as an effective model for implementation in other regions of the country and neighboring countries.

### 5.1. Limitations

This study has several limitations that should be considered when interpreting the findings. First, the surveillance program primarily relied on conventional larval and oviposition‐based entomological indices (HI, CI, BI, and egg index), which may not always accurately reflect the actual risk of dengue transmission. Adult mosquito surveillance methods were not implemented during the study period due to limitations in human and financial resources; therefore, adult vector density, which may provide a more direct estimate of transmission risk, could not be assessed.

Second, entomological inspections were conducted by multiple field teams involved in routine public health activities. Although personnel were trained and worked under standardized surveillance protocols, variations in field experience and skill levels may have introduced observer‐related bias in the detection of breeding sites and immature stages.

Third, the presence of cryptic or hidden breeding habitats that are difficult to identify during routine household inspections may have led to an underestimation of the true vector population and entomological indices. Finally, variations in surveillance capacity over time, including changes in the number of field staff and operational resources, may have influenced the intensity of sampling and the number of inspected households. These factors should be taken into account when interpreting the temporal trends reported in this study.

## 6. Conclusion

The experience gained from this study, including the effective intra and intersectoral collaboration, community engagement, and awareness‐raising initiatives, can serve as a valuable model for other regions in Iran and neighboring countries facing similar challenges. In 2024, the first local transmission of the disease, with 12 cases, was reported from the Bandar‐Lengeh region located in the vicinity of Bandar Abbas County. Therefore, local health systems must implement appropriate DF control measures even when the entomological indices are low because the conventional indices rate is largely influenced by the quality and quantity of health workers, as well as the cooperation of local residents in vector control programs. Moreover, in newly affected areas, there are cryptic breeding habitats that are not easy to identify, and over time, they will gradually be able to find them. This issue will affect both the level of these indices and the number of disease cases.

## Author Contributions

Madineh Abbasi and Saideh Yousefi contributed to the conceptualization, methodology, and drafting of the original manuscript. Abdoljabbar Zakeri, Hossein Farshidi, Mahmoud Hosseinpour, Yousef Salari, Jafar Hatami, Kaveh Soleimani, and Masoud Yaryan contributed to data collection and analysis. Fatemeh Bagheri supervised the study and critically reviewed the manuscript. Fatemeh Nikpour, Abdolreza Miroliaei, Ahmad Raeisi, Ahmadali Enayati, Mohammad Mehdi Sedaghat, and Ghobad Moradi contributed to the final editing of the manuscript.

## Funding

This study was funded by a grant from Hormozgan University of Medical Sciences (No. 4030170).

## Disclosure

All authors have read and approved the final version of the manuscript and agree to be accountable for all aspects of the work and consent to its publication.

## Ethics Statement

This study was approved by the Ethical Committee of Hormozgan University of Medical Sciences (approval no. IR.HUMS.REC.1403.362). As this was a retrospective analysis of routinely collected surveillance data, informed consent from individual participants was waived.

## Conflicts of Interest

The authors declare no conflicts of interest.

## Data Availability

The data that support the findings of this study are available from the corresponding author upon reasonable request.

## References

[1] Janjoter S. , Kataria D. , Yadav M. , Dahiya N. , and Sehrawat N. , Transovarial Transmission of mosquito-borne Viruses: a Systematic Review, Frontiers in Cellular and Infection Microbiology. (2024) 13, 10.3389/fcimb.2023.1304938. PMC10791847 38235494

[2] Mukhopadhyay K. , Sengupta M. , Misra S. C. , and Majee K. , Trends in Emerging vector-borne Viral Infections and Their Outcome in Children over Two Decades, Pediatric Research. (2024) 95, no. 2, 464–479, 10.1038/s41390-023-02866-x. 37880334

[3] Shaibu J. O. , Akinyemi K. O. , Uzor O. H. , Audu R. A. , and Bola Oriowo Oyefolu A. , Molecular Surveillance of Arboviruses in Nigeria, BMC Infectious Diseases. (2023) 23, no. 1, 10.1186/s12879-023-08526-z. PMC10436428 37596550

[4] Nadjib M. , Setiawan E. , Putri S. et al., Economic Burden of Dengue in Indonesia, PLoS Neglected Tropical Diseases. (2019) 13, no. 1, 10.1371/journal.pntd.0007038. PMC6343936 30629593

[5] Shepard D. S. , Undurraga E. A. , Halasa Y. A. , and Stanaway J. D. , The Global Economic Burden of Dengue: a Systematic Analysis, Lancet Infectious Diseases. (2016) 16, no. 8, 935–941, 10.1016/s1473-3099(16)00146-8. 27091092

[6] Kala M. P. , St John A. L. , and Rathore A. P. , Dengue: Update on Clinically Relevant Therapeutic Strategies and Vaccines, Current Treatment Options in Infectious Diseases. (2023) 15, no. 2, 27–52, 10.1007/s40506-023-00263-w. 37124673 PMC10111087

[7] Diouf B. , Sene N. M. , Ndiaye E. H. et al., Resting Behavior of blood-fed Females and Host Feeding Preferences of Aedes aegypti (Diptera: Culicidae) Morphological Forms in Senegal, Journal of Medical Entomology. (2021) 58, no. 6, 2467–2473, 10.1093/jme/tjab111. 34165556

[8] Ndenga B. A. , Mutuku F. M. , Ngugi H. N. et al., Night Time Extension of *Aedes aegypti* Human Blood Seeking Activity, The American Journal of Tropical Medicine and Hygiene. (2022) 107, no. 1, 208–210, 10.4269/ajtmh.21-0309. 35640647 PMC9294705

[9] Kolimenakis A. , Heinz S. , Wilson M. L. et al., The Role of Urbanisation in the Spread of Aedes Mosquitoes and the Diseases They transmit—A Systematic Review, PLoS Neglected Tropical Diseases. (2021) 15, no. 9, 10.1371/journal.pntd.0009631. PMC8428665 34499653

[10] George A. M. , Ansumana R. , de Souza D. K. , Niyas V. K. M. , Zumla A. , and Bockarie M. J. , Climate Change and the Rising Incidence of vector-borne Diseases Globally, International Journal of Infectious Diseases. (2024) 139, 143–145, 10.1016/j.ijid.2023.12.004. 38096974

[11] Vaux A. G. and Medlock J. M. , Current Status of Invasive Mosquito Surveillance in the UK, Parasites & Vectors. (2015) 8, no. 1, 10.1186/s13071-015-0936-9. PMC4491199 26122427

[12] Organization W. H. , Dengue and Severe Dengue, 2024, World Health Organization, https://www.who.int/news-room/fact-sheets/detail/dengue-and-severe-dengue.

[13] Liu Q. M. , Gong Z. Y. , and Wang Z. , A Review of the Surveillance Techniques for *Aedes albopictus* , American Journal of Tropical Medicine and Hygiene. (2023) 108, no. 2, 245–251, 10.4269/ajtmh.20-0781. 36315996 PMC9896331

[14] Markwardt R. and Sorosjinda-Nunthawarasilp P. , Innovations in the Entomological Surveillance of Vector-borne Diseases, 2021, Cambridge Scholars Publisher.

[15] Calderón-Peláez M.-A. , Mantilla-Granados J. S. , Velandia-Romero M. L. , Calvo E. , Castellanos J. E. , and Olano V. A. , A Strategy for entomo-virological Surveillance of Yellow Fever, Dengue, Zika, and Chikungunya Viruses in field-collected Mosquitoes, MethodsX. (2023) 11, 10.1016/j.mex.2023.102356. PMC10493583 37701736

[16] Cdc Leishmaniasis status in Iran , National Center for Emerging and Zoonotic Infectious Diseases (NCEZID): Iran Ministry of Health and Medical Education, 2022, Centers for Disease Control and Prevention, https://icdc.behdasht.gov.ir/Leishmaniasisstatus.

[17] Azari-Hamidian S. and Harbach R. E. , Keys to the Adult Females and fourth-instar Larvae of the Mosquitoes of Iran (Diptera: Culicidae), Zootaxa. (2009) 2078, no. 1, 1–33, 10.11646/zootaxa.2078.1.1.

[18] Saghafipour A. and Khazaei M. , Surveillance and Control of Dengue Fever as a Global Human Treat, Journal of Research in Health Sciences. (2024) 24, no. 4, 10.34172/jrhs.2024.169. PMC11492528 39431659

[19] Gubler D. J. , Dengue and Dengue Hemorrhagic Fever, Clinical Microbiology Reviews. (1998) 11, no. 3, 480–496, 10.1128/cmr.11.3.480. 9665979 PMC88892

[20] Dorzaban H. , Soltani A. , Alipour H. et al., Mosquito Surveillance and the First Record of Morphological and Molecular-based Identification of Invasive Species Aedes (Stegomyia) Aegypti (Diptera: Culicidae), Southern Iran, Experimental Parasitology. (2022) 236, 10.1016/j.exppara.2022.108235. 35247382

[21] Davarpanah M. A. and Kouhi P. , A New Health Threat for Iran: Dengue Fever, Iranian Journal of Medical Sciences. (2024) 49, no. 8. 10.30476/ijms.2024.103325.3654 PMC11347595 39205817

[22] Jamal M. K. , Sanaei B. , Naderi M. et al., Investigating the Recent Outbreak of Dengue Fever in Iran: a Systematic Review, The Egyptian Journal of Internal Medicine. (2025) 37, no. 1, 1–21, 10.1186/s43162-025-00411-2.

[23] Mousavi A. , Ardalan A. , Takian A. et al., Analysis of Key Stakeholders Involved in Adaptation Measures to Tackle the Adverse Health Effects of Climate Change in the Islamic Republic of Iran: A Social Network Analysis, 2024.

[24] Abbasi M. , Nikpour F. , Rahimi S. , and Bagheri F. , Dengue Fever Epidemic and Public Health Implications in Southern Iran, Scientific Reports. (2026) 16, no. 1, 10.1038/s41598-026-39680-4. PMC12976319 41680267

[25] Abbasi M. , Noori E. S. , Mobaraki F. et al., The Emergence of Autochthonous Dengue Fever in Iran: a Comprehensive Analysis of the First Major Outbreak in Sistan and Baluchestan Province, 2024, BMC Infectious Diseases. (2026) 26, no. 1, 10.1186/s12879-026-12791-z. PMC12977486 41652392

[26] WHO. Leishmaniasis Geneva, Suitzland, 2023, World Health Organization, https://www.who.int/news-room/fact-sheets/detail/leishmaniasis.

[27] Ávila M. I. , Vajda ÉA. , Jeffrey Gutiérrez E. et al., Entomological Surveillance Planning Tool (ESPT)-Generated Actionable Evidence on Human and Vector Behaviours Optimizes Present Interventions and Reduces Exposure to Anopheles Vectors in Two Communities of Guna Yala, Panamá, Malaria Journal. (2023) 22, no. 1, 10.1186/s12936-023-04453-1. PMC9875519 36698147

[28] Subahar R. , Lubis N. S. , and Winita R. , Dengue vector Surveillance Using Vector Indices and Ovitraps in Sujung Village, 2019, Banten, Indonesia.

[29] Channa M. A. and Memon N. , Seasonal Variation in the Prevalence of Larvae of *Aedes aegypti* Mosquito in District Hyderabad, Sindh, Pakistan, Pure and Applied Biology (PAB).(2020) 9, no. 2, 1354–1363.

[30] Ahmed A. M. , Shaalan E. A. , Aboul-Soud M. A. , Tripet F. , and Al-Khedhairy A. A. , Mosquito Vectors Survey in the AL-Ahsaa District of Eastern Saudi Arabia, Journal of Insect Science. (2011) 11, no. 1, 176–11, 10.1673/031.011.17601. 22958070 PMC3462400

[31] Dieng H. , Ahmad A. H. , Mahyoub J. A. et al., Household Survey of container–breeding Mosquitoes and Climatic Factors Influencing the Prevalence of *Aedes aegypti* (Diptera: Culicidae) in Makkah City, Saudi Arabia, Asian Pacific Journal of Tropical Biomedicine. (2012) 2, no. 11, 849–857. 23569860 10.1016/S2221-1691(12)60242-1 PMC3609246

[32] Chakravarti A. and Kumaria R. , Eco-Epidemiological Analysis of Dengue Infection During an Outbreak of Dengue Fever, India, Virology Journal. (2005) 2, no. 1, 10.1186/1743-422x-2-32. PMC1087891 15831102

[33] Heriyani F. , Correlation Between Air Temperature and Humidity with the Presence of *Aedes aegypti* Larvae, Berkala Kedokteran. (2019) 15, no. 1, 1–6, 10.20527/jbk.v15i1.6086.

[34] Novitasari I. and Sugiyanto Z. , Hubungan Suhu, Kelembaban Rumah Dan Perilaku Masyarakat Tentang PSN Dan Larvasidasi Dengan Keberadaan Jentik Nyamuk Penular Demam Berdarah Dengue Di RW 01 Kelurahan Sendangguwo Semarang, 2014, Universitas Dian Nuswantoro, Semarang.

[35] Al-Azab A. , Zaituon A. , Al-Ghamdi K. , and Al-Galil F. , Surveillance of Dengue Fever Vector *Aedes aegypti* in Different Areas in Jeddah City Saudi Arabia, Advances in Animal and Veterinary Sciences. (2022) 10, no. 2, 348–353.

[36] Câmara D. C. P. , Codeço C. T. , Ayllón T. et al., Entomological Surveillance of Aedes Mosquitoes: Comparison of Different Collection Methods in an Endemic Area in Rio De Janeiro, Brazil, Tropical Medicine and Infectious Disease. (2022) 7, no. 7, 10.3390/tropicalmed7070114. PMC9324765 35878126

[37] Liu Q.-M. , Gong Z.-Y. , and Wang Z. , A Review of the Surveillance Techniques for *Aedes albopictus* , American Journal of Tropical Medicine and Hygiene. (2022) 108, no. 2, 245–251, 10.4269/ajtmh.20-0781. 36315996 PMC9896331

[38] Straetemans M. and EcgoV-RrfCVTi E. , Vector-Related Risk Mapping of the Introduction and Establishment of *Aedes albopictus* in Europe, Euro Surveillance. (2008) 13, no. 7, 7–8, 10.2807/ese.13.07.08040-en. 18445417

[39] Facchinelli L. , Koenraadt S. , Fanello C. et al., Evaluation of a Sticky Trap for Collecting Aedes (Stegomyia) Adults in a dengue-endemic Area in Thailand, American Journal of Tropical Medicine and Hygiene. (2008) 78, no. 6, 904–909, 10.4269/ajtmh.2008.78.904. 18541767

[40] Sulistyawati S. , Dwi Astuti F. , Rahmah U. S. et al., Dengue vector Control Through Community Empowerment: Lessons Learned From a Community-Based Study in Yogyakarta, Indonesia, International Journal of Environmental Research and Public Health. (2019) 16, no. 6, 10.3390/ijerph16061013. PMC6466136 30897770

[41] Borah H. and Bora D. , Ecological and Social Determinants of Aedes aegypti and Aedes albopictus Larval Habitat in Northeastern India, 2022.

[42] Basso C. , da Rosa E. G. , Lairihoy R. et al., Scaling up of an Innovative Intervention to Reduce Risk of Dengue, Chikungunya, and Zika Transmission in Uruguay in the Framework of an Intersectoral Approach with and Without Community Participation, American Journal of Tropical Medicine and Hygiene. (2017) 97, no. 5, 1428–1436, 10.4269/ajtmh.17-0061. 28820690 PMC5817745

[43] Bureau P. A. S. , Dengue and Dengue Hemorrhagic Fever in the Americas: Guidelines for Prevention and Control, 1994, Pan American Health Organization.

[44] Liu X. and Liu Q. , Aedes Surveillance and Risk Warnings for Dengue—China, 2016−2019, China CDC Weekly. (2020) 2, no. 24, 431–437, 10.46234/ccdcw2020.111. 34594673 PMC8428436

[45] Nuraeni M. , Analysis of *Aedes aegypti* Larval Density on the Potential Transmission of Dengue Fever, Eduvest-Journal of Universal Studies. (2024) 4, no. 3, 1130–1137, 10.59188/eduvest.v4i3.1105.

[46] Ferdousi F. , Yoshimatsu S. , Ma E. , Sohel N. , and Wagatsuma Y. , Identification of Essential Containers for Aedes Larval Breeding to Control Dengue in Dhaka, Bangladesh, Tropical Medicine and Health. (2015) 43, no. 4, 253–264, 10.2149/tmh.2015-16. 26865829 PMC4689612

[47] Yuliawati S. , Saripudin A. , Martini M. , Saraswati L. D. , and Hestiningsih R. , Environmental Factors and Vector Density Analysis of Dengue Haemorrhagic Fever in Rowosari Health Center, Semarang, European Journal of Molecular and Clinical Medicine. (2021) 7, no. 10, 2370–2377.

[48] Balakrishnan N. , Venkatesh S. , and Lal S. , An Entomological Study on the Dengue Vectors During Outbreak of Dengue in Tiruppur Town and Its Surroundings, Tamil Nadu, India, Journal of Communicable Diseases. (2006) 38, no. 2. 17370680

[49] Pham H. V. , Doan H. T. , Phan T. T. , and Tran Minh N. N. , Ecological Factors Associated with Dengue Fever in a Central Highlands Province, Vietnam, BMC Infectious Diseases. (2011) 11, no. 1, 10.1186/1471-2334-11-172. PMC3126728 21679398

[50] Siregar F. A. and Makmur T. , Survey on Aedes mosquito Density and Pattern Distribution of Aedes aegypti and Aedes albopictus in High and Low Incidence Districts in North Sumatera Province, *IOP Conference Series: Earth and Environmental Science*, 2018, IOP Publishing.

[51] Aziz S. , Aidil R. , Nisfariza M. et al., Spatial Density of Aedes Distribution in Urban Areas: a Case Study of Breteau Index in Kuala Lumpur, Malaysia, Journal of Vector Borne Diseases. (2014) 51, no. 2, 91–96, 10.4103/0972-9062.134805. 24947215

[52] Malik M. A. , Sajid M. S. , Al-Akeel R. K. et al., Stegomyia Indices and Pattern Recognition of *Aedes aegypti* (Diptera: Culicidae) in Selected Agrogeoclimatic Zones of Punjab, Pakistan, Saudi Journal of Biological Sciences. (2024) 31, no. 2, 10.1016/j.sjbs.2023.103919. PMC10787296 38223132

[53] Erlanger T. E. , Keiser J. , and Utzinger J. , Effect of Dengue vector Control Interventions on Entomological Parameters in Developing Countries: A Systematic Review and meta-analysis, Medical and Veterinary Entomology. (2008) 22, no. 3, 203–221, 10.1111/j.1365-2915.2008.00740.x. 18816269

[54] Morato V. C. , Teixeira M. G. , Gomes A. C. , Bergamaschi D. P. , and Barreto M. L. , Infestation of *Aedes aegypti* Estimated by Oviposition Traps in Brazil, Revista de Saúde Pública. (2005) 39, no. 4, 553–558, 10.1590/s0034-89102005000400006. 16113903

